# Silver europium(III) polyphosphate

**DOI:** 10.1107/S1600536809004085

**Published:** 2009-02-11

**Authors:** Mounir Ayadi, Mokhtar Férid, Bernard Moine

**Affiliations:** a Laboratoire d’Application de la Chimie aux Ressources et, Substances Naturelles et á l’Environnement, Faculté des Sciences de Bizerte, 7021 Zarzouna Bizerte, Tunisia; b Unité de Recherches de Matériaux de Terres Rares, Centre National de Recherches en Sciences des Matériaux, BP 95 Hammam-Lif 2050, Tunisia; c LPCML–UMR 5620 CNRS/UCBL, Domaine Scientifique de la Doua, Université Claude Bernard Lyon 1 Batiment Alfred Kastler, 10 rue André-Marie Ampére, 69622 Villeurbanne Cedex, France

## Abstract

Europium(III) silver polyphosphate, AgEu(PO_3_)_4_, was prepared by the flux method. The atomic arrangement is built up by infinite (PO_3_)_*n*_ chains (periodicity of 4) extending along the *c* axis. These chains are joined to each other by EuO_8_ dodeca­hedra. The Ag^+^ cations are located in the voids of this arrangement and are surrounded by five oxygen atoms in a distorted [4+1] coordination.

## Related literature

For isotypic AgNd(PO_3_)_4_, see: Trunov *et al.* (1990 ▶). For related structures, see: Yamada *et al.* (1974 ▶); Hashimoto *et al.* (1991 ▶); Horchani *et al.* (2003 ▶); Durif (1995 ▶); Averbuch-Pouchot & Bagieu-Beucher (1987 ▶); Férid (2006 ▶).

## Experimental

### 

#### Crystal data

AgEu(PO_3_)_4_
*M* *_r_* = 575.72
Monoclinic, 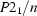

*a* = 9.9654 (3) Å
*b* = 13.1445 (7) Å
*c* = 7.2321 (3) Å
β = 90.42 (1)°
*V* = 947.31 (7) Å^3^
*Z* = 4
Mo *K*α radiation
μ = 9.37 mm^−1^
*T* = 298 (2) K
0.19 × 0.18 × 0.17 mm

#### Data collection

Nonius KappaCCD diffractometer
Absorption correction: multi-scan (*SADABS*; Sheldrick, 1996 ▶) *T*_min_ = 0.167, *T*_max_ = 0.201
3371 measured reflections
2019 independent reflections
1704 reflections with *I* > 2σ(*I*)
*R*_int_ = 0.042

#### Refinement

*R*[*F* ^2^ > 2σ(*F* ^2^)] = 0.042
*wR*(*F* ^2^) = 0.109
*S* = 1.03
2019 reflections
164 parameters
Δρ_max_ = 2.43 e Å^−3^
Δρ_min_ = −2.06 e Å^−3^

### 

Data collection: *COLLECT* (Nonius, 2001 ▶); cell refinement: *DENZO*/*SCALEPACK* (Otwinowski & Minor, 1997 ▶); data reduction: *DENZO*/*SCALEPACK*; program(s) used to solve structure: *SHELXS97* (Sheldrick, 2008 ▶); program(s) used to refine structure: *SHELXL97* (Sheldrick, 2008 ▶); molecular graphics: *DIAMOND* (Brandenburg, 1998 ▶); software used to prepare material for publication: *SHELXL97*.

## Supplementary Material

Crystal structure: contains datablocks I, global. DOI: 10.1107/S1600536809004085/br2089sup1.cif

Structure factors: contains datablocks I. DOI: 10.1107/S1600536809004085/br2089Isup2.hkl

Additional supplementary materials:  crystallographic information; 3D view; checkCIF report

## Figures and Tables

**Fig. 1.**
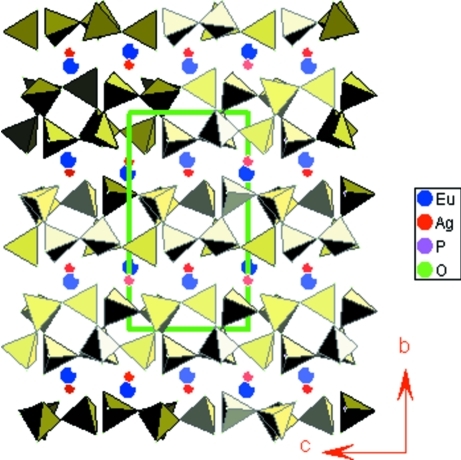
: The structural arrangement of AgEu(PO3)4 along a axis.

**Fig. 2.**
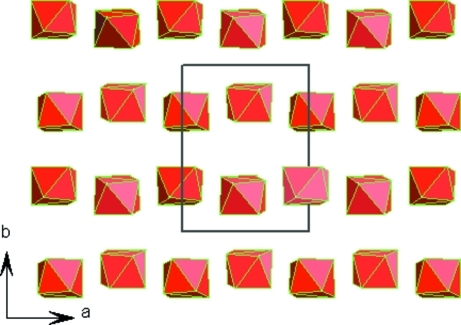
: Projection of EuO8 dodecahedra viewed along [0 0 1] direction.

**Fig. 3.**
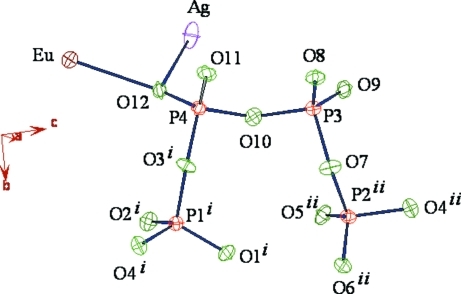
: The sequence of PO4 tetrahedra in AgEu(PO3)4, with displacement ellipsoids drawn at the 50% probability level. [Symmetry codes: (i) 1-x, 2-y, 2-z; (ii) 1-x/2, 2-y, 3-z]

## supplementary crystallographic information

### Comment

In the last decades, investigation of the synthesis and characterization of rare earth polyphosphates has gained much attention due to their potential applications in diverse areas such as phosphors and laser materials (Yamada *et al.*, 1974; Hashimoto *et al.*, 1991; Horchani *et al.*, 2003). In aim to study the condensed phosphates of rare earth and monovalent cations of general formula MILn(PO_3_)_4_ (with MI = monovalent cation)(Durif, 1995), (Ln = Eu, Er, Yb), we have synthesized single crystals of silver europium polyphosphate and investigated its crystalline structure. The atomic arrangement of this structure is characterized by a three-dimensional framework built of (PO_3_)_n_ chains that are formed by corner-sharing of PO_4_ tetrahedra. Eu3+ and Ag+ cations alternate in the middle of four such chains with Eu—Ag distances of 3.64 (7) Å (figures 1,3). The EuO_8_ dodecahedra are isolated from each other and the distances Eu—O are arranged in interval 2.355 (5)- 2.508 (5)Å (figure 2). The polyphosphate chains display two types of distances, P—O terminal ranging from 1.479 (5)  to 1.505 (5)Å  and P—O bridging, ranging from 1.585 (5)to 1.609 (5)Å. These distances are comparable with those repoted for other condensed phosphates (Durif, 1995; Averbuch-Pouchot & Bagieu Beucher, 1987; Férid (2006). The structural study reported for silver neodymium polyphosphate AgNd(PO_3_)_4_ (Trunov *et al.*,  1990) showed that the compound crystallize in the P2_1_/n space group and has similar unit cell prameters compared to AgEu(PO_3_)_4_.

### Experimental

Single crystals of AgEu(PO_3_)_4_ were prepared by flux method. A mixture of Ag_2_CO_3_ (3 g), EuCl_3.6_H_2_O (0.5 g) and H_3_PO_4_(85%, 17 ml), was progressively heated in a vitreous carbon crucible to 473 K for 12 h. The temperature was then raised and kept at 600 K for 16 days after that, the furnance was slowly cooled until the room temperature. The product was washed with boiling water to separate colorless single crystals from phosphoric acid.

### Refinement

The highest peak and the deepest hole are located 1.22Å and 0.73 Å, respectively, from Ag and Eu.

### Crystal data

**Table:**

AgEu(PO_3_)_4_	*F*(000) = 1064
*M**_r_* = 575.72	*D*_x_ = 4.037 Mg m^−^^3^
Monoclinic, *P*2_1_/*n*	Mo *K*α radiation, λ = 0.71073 Å
Hall symbol: -P 2yn	Cell parameters from 25 reflections
*a* = 9.9654 (3) Å	θ = 2.4–30.1°
*b* = 13.1445 (7) Å	µ = 9.37 mm^−^^1^
*c* = 7.2321 (3) Å	*T* = 298 K
β = 90.42 (1)°	Prism, colorless
*V* = 947.31 (7) Å^3^	0.19 × 0.18 × 0.17 mm
*Z* = 4	

### Data collection

**Table:**

Nonius KappaCCD diffractometer	2019 independent reflections
Radiation source: fine-focus sealed tube	1704 reflections with *I* > 2σ(*I*)
graphite	*R*_int_ = 0.042
φ and ω scans	θ_max_ = 27.5°, θ_min_ = 3.8°
Absorption correction: multi-scan (*SADABS*; Sheldrick, 1996)	*h* = −12→12
*T*_min_ = 0.167, *T*_max_ = 0.201	*k* = −16→15
3371 measured reflections	*l* = −9→9

### Refinement

**Table:**

Refinement on *F*^2^	Primary atom site location: structure-invariant direct methods
Least-squares matrix: full	Secondary atom site location: difference Fourier map
*R*[*F*^2^ > 2σ(*F*^2^)] = 0.042	*w* = 1/[σ^2^(*F*_o_^2^) + (0.0685*P*)^2^ + 1.0941*P*] where *P* = (*F*_o_^2^ + 2*F*_c_^2^)/3
*wR*(*F*^2^) = 0.109	(Δ/σ)_max_ < 0.001
*S* = 1.03	Δρ_max_ = 2.42 e Å^−^^3^
2019 reflections	Δρ_min_ = −2.06 e Å^−^^3^
164 parameters	Extinction correction: *SHELXL08* (Sheldrick, 2008), Fc^*^=kFc[1+0.001xFc^2^λ^3^/sin(2θ)]^-1/4^
0 restraints	Extinction coefficient: 0.00014 (2)

### Special details

**Table:**

Geometry. All e.s.d.'s (except the e.s.d. in the dihedral angle between two l.s. planes) are estimated using the full covariance matrix. The cell e.s.d.'s are taken into account individually in the estimation of e.s.d.'s in distances, angles and torsion angles; correlations between e.s.d.'s in cell parameters are only used when they are defined by crystal symmetry. An approximate (isotropic) treatment of cell e.s.d.'s is used for estimating e.s.d.'s involving l.s. planes.
Refinement. Refinement of *F*^2^ against ALL reflections. The weighted *R*-factor *wR* and goodness of fit *S* are based on *F*^2^, conventional *R*-factors *R* are based on *F*, with *F* set to zero for negative *F*^2^. The threshold expression of *F*^2^ > σ(*F*^2^) is used only for calculating *R*-factors(gt) *etc*. and is not relevant to the choice of reflections for refinement. *R*-factors based on *F*^2^ are statistically about twice as large as those based on *F*, and *R*- factors based on ALL data will be even larger.

### Fractional atomic coordinates and isotropic or equivalent isotropic displacement parameters (Å^2^)

**Table:**

	*x*	*y*	*z*	*U*_iso_*/*U*_eq_	
Eu	0.52254 (4)	0.78212 (2)	0.51227 (4)	0.01862 (19)	
Ag	0.43355 (7)	0.77670 (5)	1.00017 (8)	0.0322 (2)	
P1	0.25190 (18)	0.90008 (12)	1.2536 (2)	0.0168 (4)	
P2	0.19511 (18)	0.87338 (12)	1.6486 (2)	0.0167 (4)	
P3	0.79921 (18)	0.90983 (12)	1.2635 (2)	0.0172 (4)	
P4	0.73739 (18)	0.88594 (12)	0.8738 (2)	0.0162 (4)	
O1	0.1995 (5)	0.8356 (3)	1.1018 (6)	0.0241 (11)	
O2	0.3997 (5)	0.8951 (4)	1.2904 (7)	0.0243 (11)	
O3	0.2039 (5)	1.0133 (3)	1.2168 (7)	0.0240 (11)	
O4	0.1629 (5)	0.8769 (3)	1.4335 (6)	0.0198 (10)	
O5	0.3444 (5)	0.8678 (4)	1.6767 (7)	0.0218 (10)	
O6	0.1062 (6)	0.7906 (3)	1.7231 (6)	0.0228 (11)	
O7	0.8644 (5)	1.0214 (3)	1.2777 (7)	0.0193 (10)	
O8	0.9144 (5)	0.8395 (3)	1.2329 (6)	0.0236 (10)	
O9	0.7055 (5)	0.8919 (3)	1.4217 (6)	0.0218 (10)	
O10	0.7085 (5)	0.9202 (3)	1.0822 (6)	0.0203 (10)	
O11	0.8483 (5)	0.8097 (3)	0.8671 (7)	0.0218 (10)	
O12	0.6047 (5)	0.8557 (3)	0.7933 (6)	0.0198 (10)	

### Atomic displacement parameters (Å^2^)

**Table:**

	*U*^11^	*U*^22^	*U*^33^	*U*^12^	*U*^13^	*U*^23^
Eu	0.0183 (3)	0.0189 (2)	0.0186 (2)	−0.00063 (12)	−0.00048 (15)	−0.00108 (12)
Ag	0.0262 (4)	0.0456 (4)	0.0249 (3)	0.0034 (2)	0.0008 (2)	−0.0088 (2)
P1	0.0201 (9)	0.0138 (7)	0.0165 (7)	−0.0001 (6)	0.0005 (6)	0.0001 (6)
P2	0.0173 (9)	0.0155 (8)	0.0173 (8)	0.0007 (6)	−0.0001 (6)	−0.0002 (6)
P3	0.0206 (9)	0.0143 (7)	0.0168 (8)	0.0004 (6)	−0.0017 (6)	−0.0002 (6)
P4	0.0162 (9)	0.0160 (8)	0.0165 (8)	−0.0001 (6)	−0.0020 (6)	−0.0012 (6)
O1	0.034 (3)	0.019 (2)	0.019 (2)	0.004 (2)	−0.002 (2)	−0.0060 (19)
O2	0.023 (3)	0.026 (2)	0.023 (2)	0.000 (2)	−0.002 (2)	−0.002 (2)
O3	0.028 (3)	0.016 (2)	0.028 (3)	0.007 (2)	−0.004 (2)	0.0052 (19)
O4	0.019 (3)	0.025 (2)	0.016 (2)	0.0000 (19)	−0.0004 (18)	0.0039 (18)
O5	0.017 (3)	0.024 (2)	0.025 (2)	0.0021 (19)	−0.003 (2)	−0.007 (2)
O6	0.029 (3)	0.021 (2)	0.018 (2)	−0.005 (2)	0.000 (2)	0.0038 (18)
O7	0.013 (2)	0.014 (2)	0.031 (3)	−0.0005 (18)	0.0063 (19)	−0.003 (2)
O8	0.027 (3)	0.018 (2)	0.026 (2)	0.003 (2)	−0.001 (2)	−0.002 (2)
O9	0.027 (3)	0.023 (2)	0.016 (2)	−0.004 (2)	0.0040 (19)	−0.0026 (18)
O10	0.017 (2)	0.020 (2)	0.024 (2)	0.0009 (19)	−0.0016 (18)	−0.0023 (19)
O11	0.022 (3)	0.018 (2)	0.025 (2)	0.007 (2)	−0.001 (2)	0.002 (2)
O12	0.023 (3)	0.021 (2)	0.015 (2)	0.005 (2)	−0.0053 (19)	−0.0005 (18)

### Geometric parameters (Å, °)

**Table:**

Eu—O11^i^	2.355 (5)	P2—O4	1.587 (5)
Eu—O12	2.390 (4)	P2—O7^vi^	1.598 (5)
Eu—O9^ii^	2.420 (5)	P3—O8	1.492 (5)
Eu—O5^ii^	2.422 (5)	P3—O9	1.500 (5)
Eu—O1^iii^	2.430 (5)	P3—O10	1.593 (5)
Eu—O6^iv^	2.451 (5)	P3—O7	1.607 (5)
Eu—O2^ii^	2.500 (5)	P4—O11	1.493 (5)
Eu—O8^i^	2.508 (5)	P4—O12	1.495 (5)
Eu—Ag	3.6453 (7)	P4—O3^vii^	1.592 (5)
Eu—Ag^ii^	3.8025 (7)	P4—O10	1.602 (5)
Ag—O8^i^	2.470 (5)	O1—Eu^viii^	2.430 (5)
Ag—O12	2.503 (5)	O2—Eu^v^	2.500 (5)
Ag—O6^iii^	2.511 (5)	O3—P4^vii^	1.592 (5)
Ag—O1	2.570 (5)	O5—Eu^v^	2.422 (5)
Ag—Eu^v^	3.8025 (7)	O6—Eu^ix^	2.451 (5)
P1—O1	1.479 (5)	O6—Ag^viii^	2.511 (5)
P1—O2	1.496 (6)	O7—P2^vi^	1.598 (5)
P1—O3	1.585 (5)	O8—Ag^x^	2.470 (5)
P1—O4	1.609 (5)	O8—Eu^x^	2.508 (5)
P2—O5	1.502 (5)	O9—Eu^v^	2.420 (5)
P2—O6	1.505 (5)	O11—Eu^x^	2.355 (5)
			
O11^i^—Eu—O12	146.43 (17)	O12—Ag—Eu	40.66 (10)
O11^i^—Eu—O9^ii^	137.70 (16)	O6^iii^—Ag—Eu	117.27 (12)
O12—Eu—O9^ii^	74.62 (16)	O1—Ag—Eu	119.92 (10)
O11^i^—Eu—O5^ii^	85.21 (16)	O8^i^—Ag—Eu^v^	142.17 (11)
O12—Eu—O5^ii^	69.01 (16)	O12—Ag—Eu^v^	114.81 (10)
O9^ii^—Eu—O5^ii^	114.34 (16)	O6^iii^—Ag—Eu^v^	39.40 (11)
O11^i^—Eu—O1^iii^	108.89 (17)	O1—Ag—Eu^v^	85.49 (10)
O12—Eu—O1^iii^	77.74 (15)	Eu—Ag—Eu^v^	152.34 (2)
O9^ii^—Eu—O1^iii^	84.55 (16)	O1—P1—O2	116.6 (3)
O5^ii^—Eu—O1^iii^	134.29 (16)	O1—P1—O3	108.0 (3)
O11^i^—Eu—O6^iv^	70.99 (18)	O2—P1—O3	111.5 (3)
O12—Eu—O6^iv^	140.07 (18)	O1—P1—O4	107.3 (3)
O9^ii^—Eu—O6^iv^	74.93 (16)	O2—P1—O4	113.3 (3)
O5^ii^—Eu—O6^iv^	148.82 (17)	O3—P1—O4	98.4 (3)
O1^iii^—Eu—O6^iv^	74.24 (16)	O5—P2—O6	120.1 (3)
O11^i^—Eu—O2^ii^	70.26 (16)	O5—P2—O4	109.1 (3)
O12—Eu—O2^ii^	117.94 (15)	O6—P2—O4	104.9 (3)
O9^ii^—Eu—O2^ii^	80.70 (17)	O5—P2—O7^vi^	111.5 (3)
O5^ii^—Eu—O2^ii^	71.45 (17)	O6—P2—O7^vi^	106.6 (3)
O1^iii^—Eu—O2^ii^	154.17 (16)	O4—P2—O7^vi^	103.2 (3)
O6^iv^—Eu—O2^ii^	81.49 (16)	O8—P3—O9	120.0 (3)
O11^i^—Eu—O8^i^	68.78 (16)	O8—P3—O10	111.3 (3)
O12—Eu—O8^i^	82.12 (15)	O9—P3—O10	106.8 (3)
O9^ii^—Eu—O8^i^	151.72 (16)	O8—P3—O7	105.3 (3)
O5^ii^—Eu—O8^i^	70.38 (16)	O9—P3—O7	110.4 (3)
O1^iii^—Eu—O8^i^	74.87 (16)	O10—P3—O7	101.6 (3)
O6^iv^—Eu—O8^i^	116.40 (15)	O11—P4—O12	117.5 (3)
O2^ii^—Eu—O8^i^	125.20 (17)	O11—P4—O3^vii^	105.7 (3)
O11^i^—Eu—Ag	103.74 (12)	O12—P4—O3^vii^	112.8 (3)
O12—Eu—Ag	43.03 (12)	O11—P4—O10	111.0 (3)
O9^ii^—Eu—Ag	117.57 (11)	O12—P4—O10	106.1 (3)
O5^ii^—Eu—Ag	49.41 (11)	O3^vii^—P4—O10	102.9 (3)
O1^iii^—Eu—Ag	84.88 (11)	P1—O1—Eu^viii^	144.5 (3)
O6^iv^—Eu—Ag	154.81 (10)	P1—O1—Ag	93.9 (3)
O2^ii^—Eu—Ag	120.77 (12)	Eu^viii^—O1—Ag	112.98 (17)
O8^i^—Eu—Ag	42.51 (10)	P1—O2—Eu^v^	128.1 (3)
O11^i^—Eu—Ag^ii^	52.44 (12)	P1—O3—P4^vii^	137.7 (4)
O12—Eu—Ag^ii^	155.75 (11)	P2—O4—P1	133.6 (3)
O9^ii^—Eu—Ag^ii^	85.32 (11)	P2—O5—Eu^v^	133.2 (3)
O5^ii^—Eu—Ag^ii^	108.69 (11)	P2—O6—Eu^ix^	142.4 (3)
O1^iii^—Eu—Ag^ii^	114.28 (11)	P2—O6—Ag^viii^	115.3 (2)
O6^iv^—Eu—Ag^ii^	40.55 (12)	Eu^ix^—O6—Ag^viii^	100.06 (18)
O2^ii^—Eu—Ag^ii^	43.64 (11)	P2^vi^—O7—P3	131.3 (3)
O8^i^—Eu—Ag^ii^	120.64 (11)	P3—O8—Ag^x^	108.8 (3)
Ag—Eu—Ag^ii^	152.34 (2)	P3—O8—Eu^x^	145.5 (3)
O8^i^—Ag—O12	80.67 (15)	Ag^x^—O8—Eu^x^	94.16 (15)
O8^i^—Ag—O6^iii^	109.46 (15)	P3—O9—Eu^v^	140.7 (3)
O12—Ag—O6^iii^	93.64 (16)	P3—O10—P4	130.2 (3)
O8^i^—Ag—O1	110.19 (16)	P4—O11—Eu^x^	150.8 (3)
O12—Ag—O1	132.06 (14)	P4—O12—Eu	137.3 (3)
O6^iii^—Ag—O1	122.79 (16)	P4—O12—Ag	118.8 (2)
O8^i^—Ag—Eu	43.33 (11)	Eu—O12—Ag	96.31 (16)

Symmetry codes: (i) *x*−1/2, −*y*+3/2, *z*−1/2; (ii) *x*, *y*, *z*−1; (iii) *x*+1/2, −*y*+3/2, *z*−1/2; (iv) *x*+1/2, −*y*+3/2, *z*−3/2; (v) *x*, *y*, *z*+1; (vi) −*x*+1, −*y*+2, −*z*+3; (vii) −*x*+1, −*y*+2, −*z*+2; (viii) *x*−1/2, −*y*+3/2, *z*+1/2; (ix) *x*−1/2, −*y*+3/2, *z*+3/2; (x) *x*+1/2, −*y*+3/2, *z*+1/2.
